# Amorphous TiO_2_ nano-coating on stainless steel to improve its biological response

**DOI:** 10.1088/1748-605X/ad6dc4

**Published:** 2024-08-21

**Authors:** Victor I Garcia-Perez, Kelly M Hotchkiss, Phaedra Silva-Bermudez, Miryam Martínez Hernández, Gina Prado-Prone, Rene Olivares-Navarrete, Sandra E Rodil, Argelia Almaguer-Flores

**Affiliations:** 1 Laboratorio de Biointerfases, División de Estudios de Posgrado e Investigación, Facultad de Odontología, Universidad Nacional Autónoma de México. Circuito exterior s/n, Ciudad Universitaria, Ciudad de México, CDMX 04510, Mexico; 2 Department of Biomedical Engineering Commonwealth, College of Engineering, Virginia University, Richmond, VA 23284, United States of America; 3 Unidad de Ingeniería de Tejidos,Terapia Celular y Medicina Regenerativa, Instituto Nacional de Rehabilitación Luis Guillermo Ibarra Ibarra. Calzada México-Xochimilco, Ciudad de México 14389, Mexico; 4 Instituto de Investigaciones en Materiales, Universidad Nacional Autónoma de México. Circuito exterior s/n, Ciudad Universitaria, Ciudad de México, CDMX 04510, Mexico

**Keywords:** nanocoating, osteogenesis, antibacterial surfaces, inflammatory cytokines, orthopaedic implants

## Abstract

This study delves into the potential of amorphous titanium oxide (aTiO_2_) nano-coating to enhance various critical aspects of non-Ti-based metallic orthopedic implants. These implants, such as medical-grade stainless steel (SS), are widely used for orthopedic devices that demand high strength and durability. The aTiO_2_ nano-coating, deposited via magnetron sputtering, is a unique attempt to improve the osteogenesis, the inflammatory response, and to reduce bacterial colonization on SS substrates. The study characterized the nanocoated surfaces (SS-a TiO_2_) in topography, roughness, wettability, and chemical composition. Comparative samples included uncoated SS and sandblasted/acid-etched Ti substrates (Ti). The biological effects were assessed using human mesenchymal stem cells (MSCs) and primary murine macrophages. Bacterial tests were carried out with two aerobic pathogens (*S. aureus* and *S. epidermidis*) and an anaerobic bacterial consortium representing an oral dental biofilm. Results from this study provide strong evidence of the positive effects of the aTiO_2_ nano-coating on SS surfaces. The coating enhanced MSC osteoblastic differentiation and exhibited a response similar to that observed on Ti surfaces. Macrophages cultured on aTiO_2_ nano-coating and Ti surfaces showed comparable anti-inflammatory phenotypes. Most significantly, a reduction in bacterial colonization across tested species was observed compared to uncoated SS substrates, further supporting the potential of aTiO_2_ nano-coating in biomedical applications. The findings underscore the potential of magnetron-sputtering deposition of aTiO_2_ nano-coating on non-Ti metallic surfaces such as medical-grade SS as a viable strategy to enhance osteoinductive factors and decrease pathogenic bacterial adhesion. This could significantly improve the performance of metallic-based biomedical devices beyond titanium.

## 1. Introduction

The success of bone-dwelling devices such as dental and craniofacial implants relies on their osseointegration potential [1–3] and the absence of device-associated infections [4, 5]. Titanium (Ti) and Ti-based alloys remain the ‘gold standard’ materials for orthopedic and dental implants due to their biocompatibility, chemical stability, and osseointegration capability [6, 7]. Several studies have established that Ti surface properties, such as surface energy or wettability, are mainly determined by the native oxide layer [8–10]. This, along with the surface roughness, influences important cellular functions like adhesion, proliferation, differentiation, and secretion of angiogenic factors in the cellular microenvironment, directly affecting the osseointegration and bone growth in the peri-implant region [11]. On the other hand, stainless steel (SS) offers acceptable mechanical properties, biocompatibility, an easy manufacturing process, and greater availability than Ti [12]. However, SS implants have reduced osseointegration due to the growth of connective fibrous tissue at the bone-implant interface, known as the foreign body response (FBR), limiting its use in load-bearing applications [13]. The FBR is driven by the inflammatory response after implantation. In the wound healing process around the implant, macrophages play an essential role in controlling the microenvironment with the production of anti-inflammatory mediators facilitating the resolution of the inflammatory response and the healing process with faster osseointegration [14] or creating a pro-inflammatory microenvironment that evolves into a biomaterial fibrous encapsulation [15]. Moreover, differences in the clinical performance of Ti implants compared to SS-based implants have been associated with their different corrosion behaviors, surface properties, toxicity by metal ions released, and infection susceptibility [16]. In this sense, medical-grade SS implants have a greater predisposition to corrosion than Ti implants due to their chemical nature [17].

Another critical challenge in orthopedic implants is the device-associated infection risk. After surgical implantation, bacteria can colonize the implant surface and subsequently develop a biofilm [18, 19]. Biofilms formed in natural or artificial environments are bacterial communities attached to a surface in which cells are embedded in an extracellular matrix of bacterial origin [20]. Bacterial adhesion to surfaces is the first step in biofilm formation and depends on complex interactions among the host, surface device, and microorganisms [21, 22]. Several studies have demonstrated that the implant surface’s chemical composition, surface energy, and roughness are important factors that influence bacterial adhesion [23, 24]. In this sense, implant surface modifications represent a local strategy for enhancing osseointegration and preventing implant-related infections [25–27].

In previous works, it has been shown that an amorphous Ti oxide nano-coating (aTiO_2_) deposited onto titanium surfaces significantly enhances the adhesion and osteoblastic differentiation of human mesenchymal stem cells (MSCs) and demonstrates a notable capability for mitigating bacterial adhesion when compared with the non-coated titanium substrates [28, 29]. Moreover, when studying the aTiO_2_ nano-coating deposited on Si substrates, mesenchymal cell differentiation into the osteoblastic phenotype was also observed [30]. However, the deposition of this nano-coating on other metallic substrates does not necessarily guarantee the same response, as it has been observed for magnesium alloy substrates, where the response to the aTiO_2_ nano-coating was not as expected due to different interactions occurring between the aTiO_2_ coating and the underlying substrate (Mg alloy) [31].

The present study aimed to explore the potential of the aTiO_2_ nanocoating deposited onto medical-grade SS substrates to improve its biological response, focusing on the osteoblastic differentiation, the anti-inflammatory response, and antibacterial protection.

## 2. Materials and Methods

### 2.1. Preparation of the experimental surfaces

Grade-2 commercially pure Ti disks (1 mm thick and 14 mm in diameter) were provided by the Institut Straumann AG, Basel, Switzerland. The Ti surfaces presented a rough sandblasted/acid-etched topography, which is normally used in dental implants. The disks were sterilized overnight with 25 kGy gamma irradiation as previously described [32].

SS substrates were produced from medical-grade SS (alloy 316 l La Paloma metals, SA de CV, Mexico), disks were 1 mm thick and 14 mm in diameter. To achieve a similar topography to the commercial Ti pieces, the SS disks were sandblasted with 300–500 μm SiO_2_ particles and subsequently acid-etched in sulfuric acid (H_2_SO_4_) for 60 s at room temperature (RT). Then, the SS samples were ultrasonically cleaned using acetone, isopropanol, and distilled water for 30 min in each solution, then dried at RT. The SS surfaces were sterilized in an autoclave for 30 min at 121 °C.

### 2.2. Amorphous Ti oxide nano-coatings

A magnetron sputtering system attached to a high-vacuum chamber (base pressure of 1.3 × 10^−4^ Pa), employing a high-purity Ti target with a 4 inch-diameter, was used to deposit the aTiO_2_ nano-coatings on the SS substrates to obtain SS-aTiO_2_ surfaces. The deposition conditions were optimized in previous works [28, 29]. Briefly, deposition was carried out at a working pressure of 20 mTorr and radiofrequency power of 200 W, using an atmosphere of argon (8 sccm) during 30 s and subsequently a combined atmosphere of argon and oxygen (8:2 sccm, Ar:O ratio) as sputtering gasses. To produce the TiO_2_ nano-coatings presenting an amorphous atomic structure, no external substrate heating was applied, and although the plasma itself produced heat (no higher than 100 °C) during the coating deposition, the maximum temperature achieved was not enough to induce a crystalline growth. The deposition time was set at 45 min to produce $ \approx $75 nm thick coatings, and the thickness of the aTiO_2_ nanocoating was corroborated using a contact profilometer Dektak IIA (Veeco Sloan/Dektak) for a sample deposited on crystalline Si wafer, where a thickness-measuring step was specifically created by partially masking part of the Si wafer before coating deposition.

The adhesion of the nano-coating to the SS substrate was optimized by using a buffer layer consisting of a thin (8 nm) Ti film deposited using only Ar atmosphere during the first 30 s of deposition at 100 °C. This condition was obtained after optimization, where different thicknesses and substrate temperatures were tested for the buffer layer deposition. The effectiveness of this strategy to improve adhesion was corroborated by the scratching test (Supplementary data).

Prior to all biological experiments, the SS-aTiO_2_ surfaces were sterilized in an autoclave for 30 min at 121 °C.

### 2.3. Surface characterization

The topography of the SS, SS-aTiO_2_, and Ti surfaces was examined by scanning electron microscopy (SEM; Carl Zeiss SMT Ltd, UK) at two magnifications (500X and 10 000X) using a voltage of 10.0 kV. Surface roughness was analyzed by confocal laser microscopy (CLM, Olympus America Inc., PA). The average surface roughness (Sa) of all surfaces was determined over the complete 3D surface using a 100X objective and 128 × 128 μm field of view using a LEXT OLS4000 software.

The wettability of the Ti, SS and SS-aTiO_2_ surfaces was evaluated measuring the contact angle (CA), using a goniometer (Ramé-Hart goniometer, 250-F1, NJ, USA) via the static sessile-drop technique, using distilled water drops (5 μl) at RT. The CA reported values were analyzed using the DROPimage software and represent the mean ± standard deviation of three independent measurements on three different surface samples.

The surface chemical composition of the samples was confirmed by x-ray photoelectron spectroscopy (XPS) using the Versaprobe II equipment (Physical Electronics), operated at 10^−9^ Torr using AlK*α* radiation. To minimize the impact of surface roughness on the analysis, high-resolution and survey XPS spectra were acquired only for mirror-polished substrates. Roughness on SS and SLA Ti substrates resulted in noisy spectra with significant adsorbed carbon that was difficult to remove with Ar cleaning. The same SS-aTiO_2_ surfaces were used for the scratching test.

### 2.4. Characterization of the osteogenic microenvironment produced by MSCs

Human bone marrow-derived MCSs (MSCs; Lonza, Walkersville, MD) were plated at passage 2 at a density of 10 000 cells cm^−2^ in 24-well plate on Ti, SS, SS-aTiO_2_ surfaces (*n* = 6) and cultured in MSC Growth Medium (Lonza, Walkersville, MD) for 7 d at 37 °C with 5% CO_2_ and 100% humidity. Cells on tissue culture polystyrene (TCPS) served as a control.

To assess MSC differentiation and osteogenic microenvironment, conditioned media from MSCs cultured on TCPS or metallic surfaces (SS, Ti or SS-aTiO_2_) was collected after 7 d of culture. Levels of osteocalcin, osteoprotegerin, BMP-2, BMP-4, and VEGF-A were analyzed by Enzyme-Linked Immunosorbent Assays (ELISA, R&D Systems, Minneapolis, MN) and normalized to DNA content in cell lysates (Promega QuantiFluor dsDNA System, Promega, Madison, WI). Alkaline phosphatase-specific activity (ALP) was also analyzed from cell lysates by measuring the conversion of *p*-nitrophenol from *p*-nitrophenyl phosphate by alkaline phosphatase at 37 °C and normalized to total protein content in the lysates.

### 2.5. Macrophage isolation, culture, and inflammatory microenvironment

Primary macrophages were derived from 10 week-old C57BL/6 mice (The Jackson Laboratory, Bar Harbor, ME) under the approval of the Virginia Commonwealth University Institutional Animal Care and Use Committee (AD10001108). Bone marrow cells were flushed from femurs, treated with ACK lysis buffer (Thermo Fisher Scientific, Waltham, MA), and plated in tissue culture flasks. Naïve macrophages were obtained by culturing cells in DMEM (Thermo Fisher Scientific) supplemented with 10% fetal bovine serum (Thermo Fisher Scientific), 50 U ml^−1^ penicillin–50 μg ml^−1^ streptomycin (Thermo Fisher Scientific), and 50 ng ml^−1^ macrophage colony-stimulating factor (BioLegend, San Diego, CA) for seven days. Cells were detached with Accutase (BioLegend) and plated in 24 well plates onto TCPS or metallic surfaces (SS, Ti or SS-aTiO_2_).

Conditioned media was collected from macrophages cultured on TCPS or metallic surfaces after 24 h of culture. Levels of IL-1*β*, IL-6, IL-10, IL-17A, and TNF were measured by ELISA (BioLegend) to assess macrophage inflammatory microenvironment and were normalized to DNA content in cell lysates as described above.

### 2.6. Gene expression analysis of MSCs and macrophages

To quantify gene expression of osteogenic genes in MSCs and inflammatory markers on murine macrophages, cells were plated according to the cell culture conditions described above on either TCPS or metallic surfaces (SS, Ti or SS-aTiO_2_). Macrophages were cultured for 12 h, and MSCs were cultured for 7 d and then incubated with fresh media for 12 h. After incubation, mRNA was extracted using TRIzol reagent (Thermo Fisher Scientific). cDNA templates were created with amplified RNA (500 ng) using iScript cDNA synthesis kit (Bio-Rad, Hercules, CA). Real-time quantitative Polymerase Chain Reaction (qPCR) was performed using SsoAdvanced Universal SYBR Green Supermix (Bio-Rad) to assess expression of osteogenic (RUNX2, SP7, and SPP1) or inflammatory (Arg1, Mrc1, Nos2, and Tgfb1) markers (table 1). Gene expression is presented as fold-change (2-ΔΔCt) from GAPDH housekeeping gene (Δ1) and to untreated TCPS controls (Δ2).

**Table 1.** Primer sequences used for the real-time qPCR analysis of gene expression.

Human gene	Gene symbol	Primer sequence
Glyceraldehyde 3-phosphate dehydrogenase	GAPDH	F: ATAAATTGAGCCCGCAGCC
		R: ACCAAATCCGTTGACTCCGA
Runt-related transcription factor 2	RUNX2	F: GGCGCATTTCAGATGATGA
		R: GCCCAGTTCTGAAGCACCT
Osterix	SP7	F: CATCTGCCTGGCCTCCTTG
		R: CAGGGGACTGGAGCCATA
Osteopontin	SPP1	F: GAGGGCTTGGTTGTCAGC
		R: CAATTCTCATGGTAGTGAGTTTTCC

Mouse Gene	Gene Symbol	Primer sequence

Arginase 1	Arg1	F: GAATCTGCATGGGCAACC
		R: GAATCCTGGTACATCTGGGAAC
Mannose Receptor C-Type 1 (CD206)	Mrc1	F: CCACAGCATTGAGGAGTTTG
		R: ACAGTCCATCATTTGGCTCA
Nitric Oxide Synthase 2	Nos2	F: CTTTGCCACGGACGAGAC
		R: TCATTGTACTCTGAGGGCTGAC
Transforming Growth Factor Beta 1	Tgfb1	F: TGGAGCAACATGTGGAACTC
		R: GTCAGCAGCCGGTTACCA

### 2.7. Bacterial test

The effect of nano-coating on preventing bacterial colonization was evaluated using two *in vitro* models. In the first model, single cultures of two aerobic reference strains (*S. aureus* and *S. epidermidis*) associated with implantable device infections were tested independently. In the second model, a co-culture of a consortium of eight anaerobic oral species resembling the complexity of an oral biofilm was also tested. The bacterial strains used for both experimental *in vitro* models are presented in table 2.

**Table 2.** Bacterial species used in the *in vitro* studies of bacterial colonization.

Bacterial strain	ATCC^a^	Gram stain	Atmosphere growth condition	*In vitro* model^b^
*Actinomyces israelii*	12 102	+	Anaerobic	Oral bacteria co-culture
*Aggregatibacter actinomycetemcomitans* serotype b	43 718	−	Anaerobic	
*Campylobacter rectus*	33 238	−	Anaerobic	
*Eikenella corrodens*	23 834	−	Anaerobic	
*Parvimonas micra*	33 270	−	Anaerobic	
*Porphyromonas gingivalis*	33 277	−	Anaerobic	
*Prevotella intermedia*	25 611	−	Anaerobic	
*Streptococcus sanguinis*	10 556	+	Anaerobic	
*Staphylococcus aureus*	25 923	+	Aerobic	Single culture
*Staphylococcus epidermidis*	14 990	+	Aerobic	

^a^ American Type Culture Collection (ATTC^®^), Rockville, MD, USA.

^b^ Corresponds to the *in vitro* model in which the bacterial strain was used to evaluate the bacterial colonization on the surfaces.

For the single-culture model, pure cultures of *S. aureus* or *S. epidermidis* were individually cultured on plates with Trypticase soy agar (TSA; BBL, Becton-Dickinson) enriched with 0.3 μg ml^−1^ menadione (Sigma-Aldrich) and 5 μg ml^−1^ hemin (Sigma-Aldrich) and incubated for 24 h at 37 °C under aerobic conditions. For the co-culture model, the eight anaerobic strains were cultured on plates prepared with *Mycoplasma* agar base (*Mycoplasma,* BBL, Becton-Dickinson) supplemented with 5% defibrinated sheep blood (Microlab Laboratory S.A de C.V), 5 μg ml^−1^ hemin and 0.3 μg ml^−1^ menadione, and cultured at 37 °C under anaerobic conditions (80% N_2_, 10% CO_2_ and 10% H_2_) for 5 d in an anaerobic chamber. After the incubation period, the different bacterial cultures were harvested and individually suspended in tubes contained enriched TSB broth (TSB added with 0.3 μg ml^−1^ menadione and 5 μg ml^−1^ hemin) or enriched *Mycoplasma* broth (*Mycoplasma* added with 5 μg ml^−1^ hemin and 0.3 μg ml^−1^ menadione), depending on the strains. The optical density (OD) in each tube was adjusted to 1 at *λ* = 600 nm in a spectrophotometer (BioPhotometer D30, Eppendorf) to obtain a bacterial suspension with 1 × 10^9^ cells ml^−1^. To test bacterial colonization, the sterilized surfaces were independently inoculated with 1 mL of the bacterial suspension of either *S. aureus, S. epidermidis*, or the anaerobic strains co-culture. The inoculated surfaces were incubated for 1 and 7 d at 37 °C in an orbital shaker (≈160 rpm), under aerobic atmosphere for the single-cultures and anaerobic conditions for the co-culture. All experiments were performed in triplicate. The *in situ* bacterial viability was estimated by the XTT assay (XTT, Invitrogen, USA). After incubation, the inoculated surfaces were rinsed twice with PBS to remove non-adhered bacteria and transferred to sterile well plates. Then, the surfaces were incubated for 3 h in 1 mL of enriched TSB or *Mycoplasma* broth added with 20 μl of the XTT solution. Finally, the O.D of 100 μl aliquots of the supernatants were read at *λ* = 450 nm in a microplate reader (FilterMax F5, Molecular devices), and the number of viable bacteria was calculated according to standard curves previously performed.

## 3. Statistical analysis

All data are presented as the mean ± standard error. Statistical analysis was performed using Prism8 (GraphPad Software, San Diego, CA). Data were first subjected to Shapiro–Wilk normality test. Results from this test indicate that the data were normally distributed. A one-factor, equal analysis of variance (ANOVA) was used to test the null hypothesis that group means were equal at a significance level of *α* = 0.05, with post-hoc Tukey’s HSD test for multiple comparisons.

## 4. Results

### 4.1. Surface characterization

**Figure 1.**
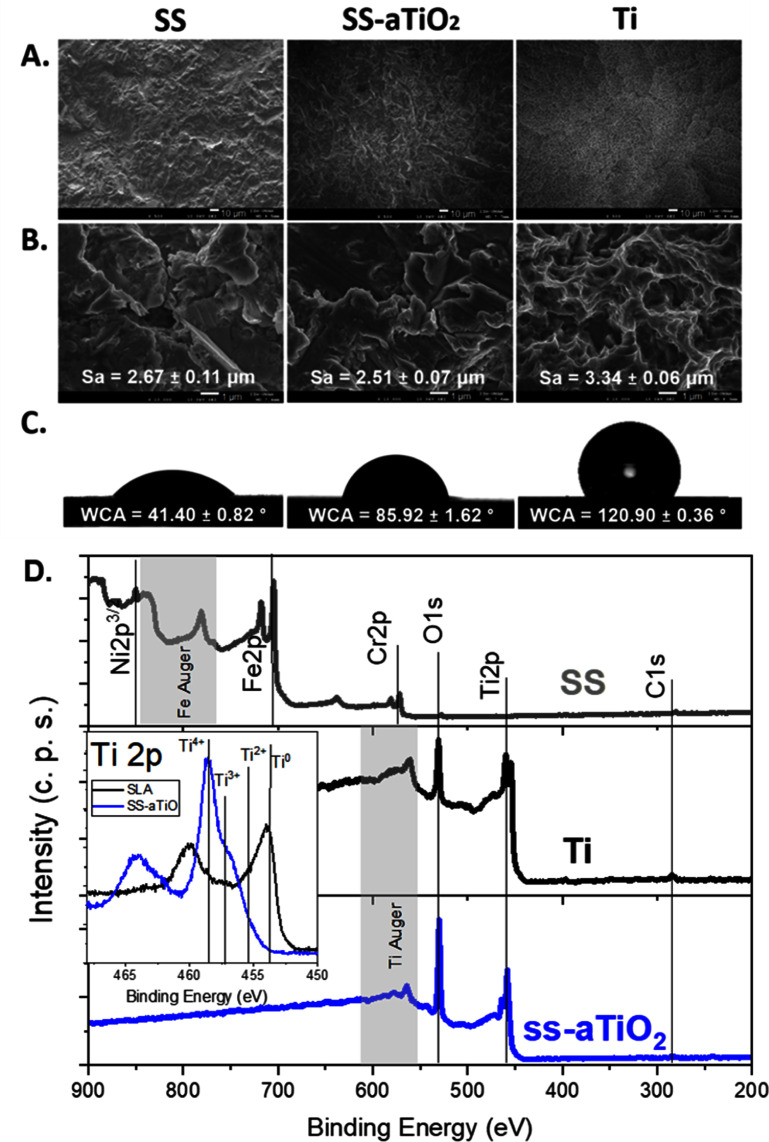
Surface characterization of medical-grade stainless steel (SS), SS surfaces modified with amorphous titanium oxide nano-coating (SS-aTiO_2_) and sandblasted/acid-etched titanium (Ti). Scanning electron images of surface topography at 500X magnification (A) and 10 000X magnification (B) with the surface roughness (Sa) determined by confocal laser microscopy. (C) Sessile drop water contact angle (WCA) values indicate the wettability of the surfaces. (D) x-ray photoelectron spectra of the three samples.

SEM, average roughness (Sa) determined by laser confocal microscopy, and water CA measurements (WCA) of the Ti, SS, and SS-aTiO_2_ surfaces are presented in figures 1(A)–(C). Profilometric measurements confirmed the amorphous TiO_2_ coating deposited on the SS substrates was ≈75 nm thick. The average roughness (Sa) of the Ti surfaces was 3.34 ± 0.06 μm, whereas the Sa of the uncoated SS and the SS-aTiO_2_ substrates was 2.67 ± 0.11 μm and 2.51 ± 0.07 μm, respectively. The treatment used to induce the roughness of the SS substrates did not produce an identical topography to that of the Ti surfaces; nevertheless, the average roughness values were in a similar range. It is important to emphasize that the nanocoating change the chemistry of the surface but did not significantly change the Sa value or microtopography, mainly due to the thin thickness of the coating and its conformal deposition as observed from the SEM images (figures (A) and (B)).

The wettability analysis revealed that the SS substrate exhibited a hydrophilic character with a WCA of 41.40° ± 0.82°; however, the deposition of the a-TiO_2_ nano-coating (SS-aTiO_2_) clearly decreased the wettability of the SS substrate surface (WCA of 85.92° ± 1.62°). While the Ti surface presented a hydrophobic character with a WCA of 120.90° ± 0.36°.

Figure 1(D) shows the XPS survey spectra of the SS, Ti, and the nano-coating, and the surface composition is reported in table 3. The signal from adsorbed carbon species was significantly reduced after cleaning the surfaces using Ar ions accelerated at 0.5 kV for 1 min at a current density of 500 nA mm^−2.^ Only C, Ti, and O signals were detected for Ti and the nano-coating. No elements from the SS substrate were detected in the SS-aTiO_2_ surfaces. The high-resolution spectra are presented after the cleaning process (Insert in figure 1(D)). The chemical shift of the Ti 2p orbitals, between the Ti surface and the nano-coating, are observed, indicating that Ti presents predominantly the Ti^4+^ oxidation state corresponding to the TiO_2_. A very small signal at lower binding energies is observed, and it was associated with a lower oxidation state, probably created during the Ar cleaning. The spectra are very similar to those reported previously for the aTiO_2_ films [28].

**Table 3.** Chemical composition in atomic concentration [at.%] of the Ti, SS, and SS-aTiO_2_ surfaces was obtained from the XPS survey spectra analysis.

	Experimental surfaces		
Element	Ti	SS	SS-aTiO_2_
O	32.3	3.2	65.7
Ti	63.1	—	29.4
C	4.6	7.0	4.8
Fe	—	72.3	—
Ni	—	3.2	—
Cr	—	13.7	—

The amorphous character of the aTiO_2_ nano-coatings was confirmed on samples deposited on a crystalline Si substrate since the 75 nm thin film on the rough SS substrate cannot be detected using grazing incidence x-ray diffraction.

### 4.2. Osteogenic differentiation and microenvironment created by the MSCs cultured on the experimental surfaces

**Figure 2.**
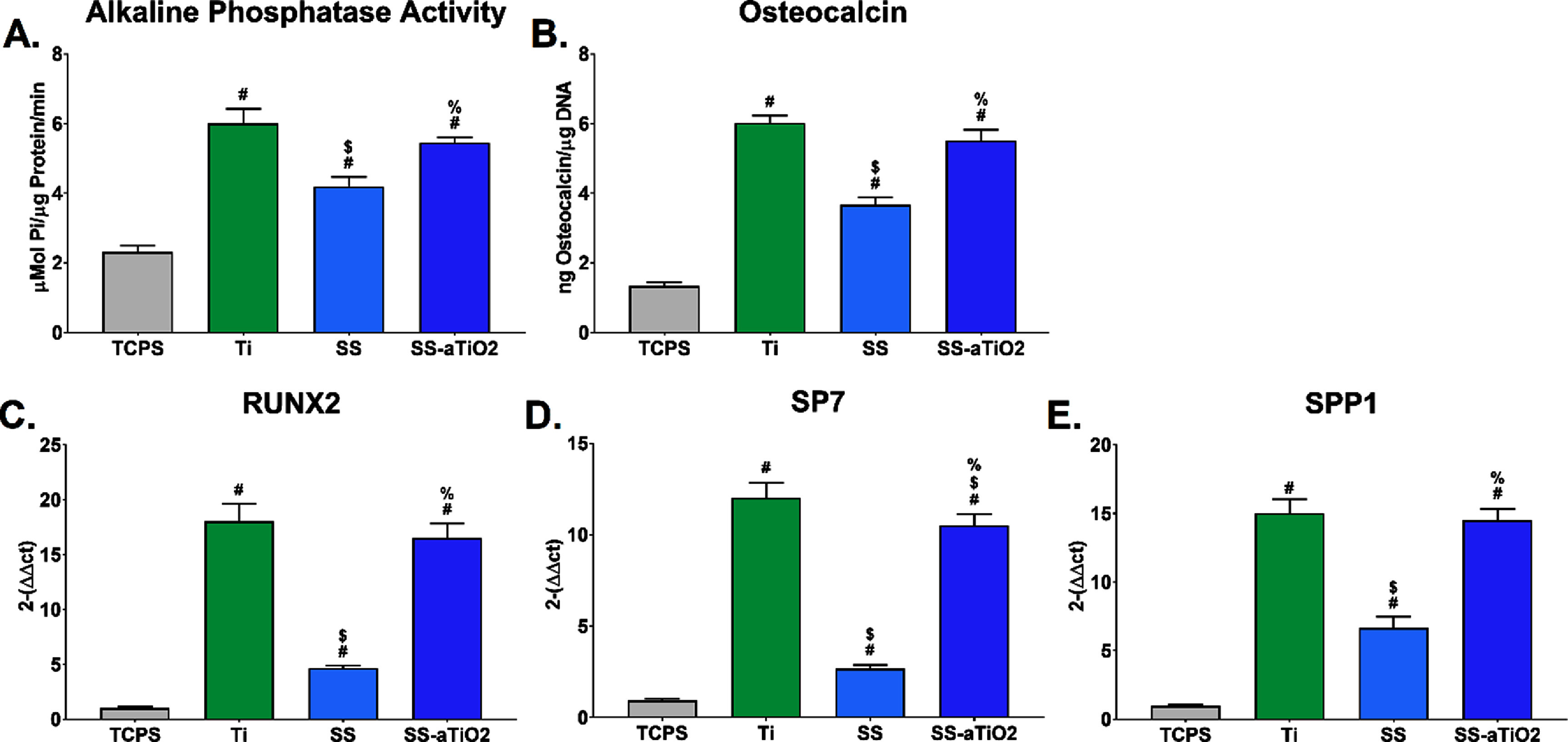
(A)–(E) Osteogenic differentiation of human mesenchymal stem cells (MSC) after 7 d of culture on the surfaces. Tissue culture polystyrene (TCPS), sandblasted/acid-etched titanium (Ti), medical-grade stainless steel (SS), and SS substrate with amorphous titanium oxide nano-coating (SS-aTiO_2_) were tested. # *p* < 0.05, surfaces *vs* TCPS; $ *p* < 0.05, surfaces *vs* Ti; % *p* < 0.05, SS *vs* SS-aTiO_2_.

To assess whether surface topography or chemistry changes induced MSCs into an osteoblastic phenotype, we measured the levels of two important molecules associated with an osteoblastic phenotype; ALP activity as an early marker and, osteocalcin as a later marker of osteoblastic differentiation. MSCs cultured on the nano-coated SS-aTiO_2_ surface exhibited significantly higher ALP activity compared to those cultured on the uncoated SS surface, and similar to ALP activity exhibited by cells cultured on the Ti surface, while the lowest ALP activity was observed in MSCs cultured on the uncoated SS surface (figure 2(A)). Furthermore, osteocalcin secretion levels were also higher in cells cultured on Ti or SS-aTiO_2_ than in cells cultured on uncoated SS surfaces (figure 2(B)). The gene expression of RUNX2 and SP7, transcription factors known as master regulators of osteogenesis, was significantly higher in MSCs cultured on metallic surfaces (SS, SS-aTiO_2_ and Ti) than in cells under TCPS control. RUNX2 and SP7 gene expression (figures 2(C) and (D)) was significantly higher on the nano-coated SS-aTiO_2_ surface when compared to the uncoated SS surface, and comparable to the gene expression in cells cultured on the Ti surface. Finally, increased levels of osteopontin gene expression (SPP1) were detected on MSCs cultured on Ti and SS-aTiO_2_ surfaces compared to SS and TCPS (figure 2(E)).

**Figure 3.**
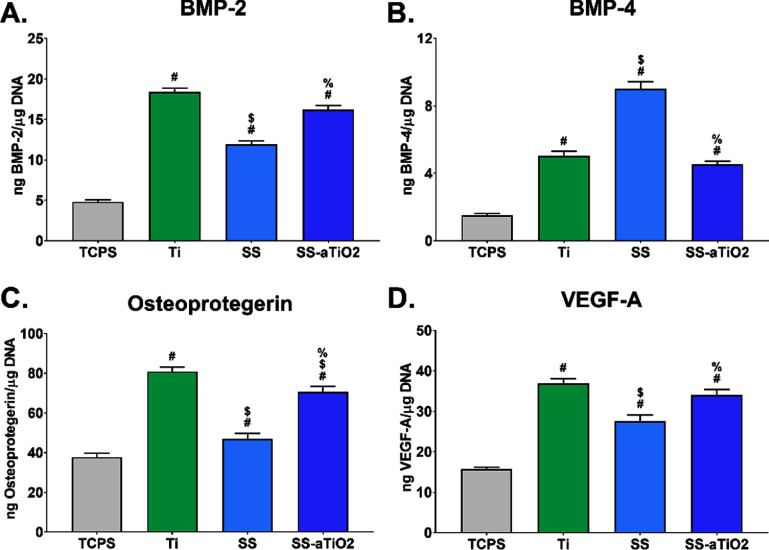
(A)–(D) Protein production of human mesenchymal stem cell (MSC) associated with bone formation, after 7 d of culture on the surfaces. Tissue culture polystyrene (TCPS), sandblasted/acid-etched titanium (Ti), medical-grade stainless steel (SS), and SS substrate with amorphous Ti oxide nano-coating (SS-aTiO_2_). # *p* < 0.05, surfaces *vs* TCPS; $ *p* < 0.05, surfaces *vs* Ti; % *p* < 0.05, SS *vs* SS-aTiO_2_.

The levels of proteins associated with bone formation in the cellular microenvironment of MSCs cultured on the experimental surfaces and TCPS control are shown in figure 3. In general, the MSCs cultured on SS-aTiO_2_ and Ti surfaces produced similar levels of these proteins. Protein secretion by MSCs was undoubtedly sensitive to the nano-coating since the levels of BMP-2, osteoprotegerin, and VEGF-A produced on the SS-aTiO_2_ surfaces were significantly increased when compared with the uncoated SS surface, and similar to those observed for cells cultured on Ti surfaces. Regarding BMP-4, reduced levels of this protein were found on the Ti and SS-aTiO_2_ surfaces in relation to the levels detected on the SS substrates. It is important to emphasize that even when nano-coating of SS surfaces reduced the expression of BMP4 compared to the uncoated SS surface, BMP4 production on SS-aTiO_2_ surfaces was similar to that on Ti surfaces.

### 4.3. Effect of the aTiO_2_ nano-coating on macrophage inflammatory microenvironment

**Figure 4.**
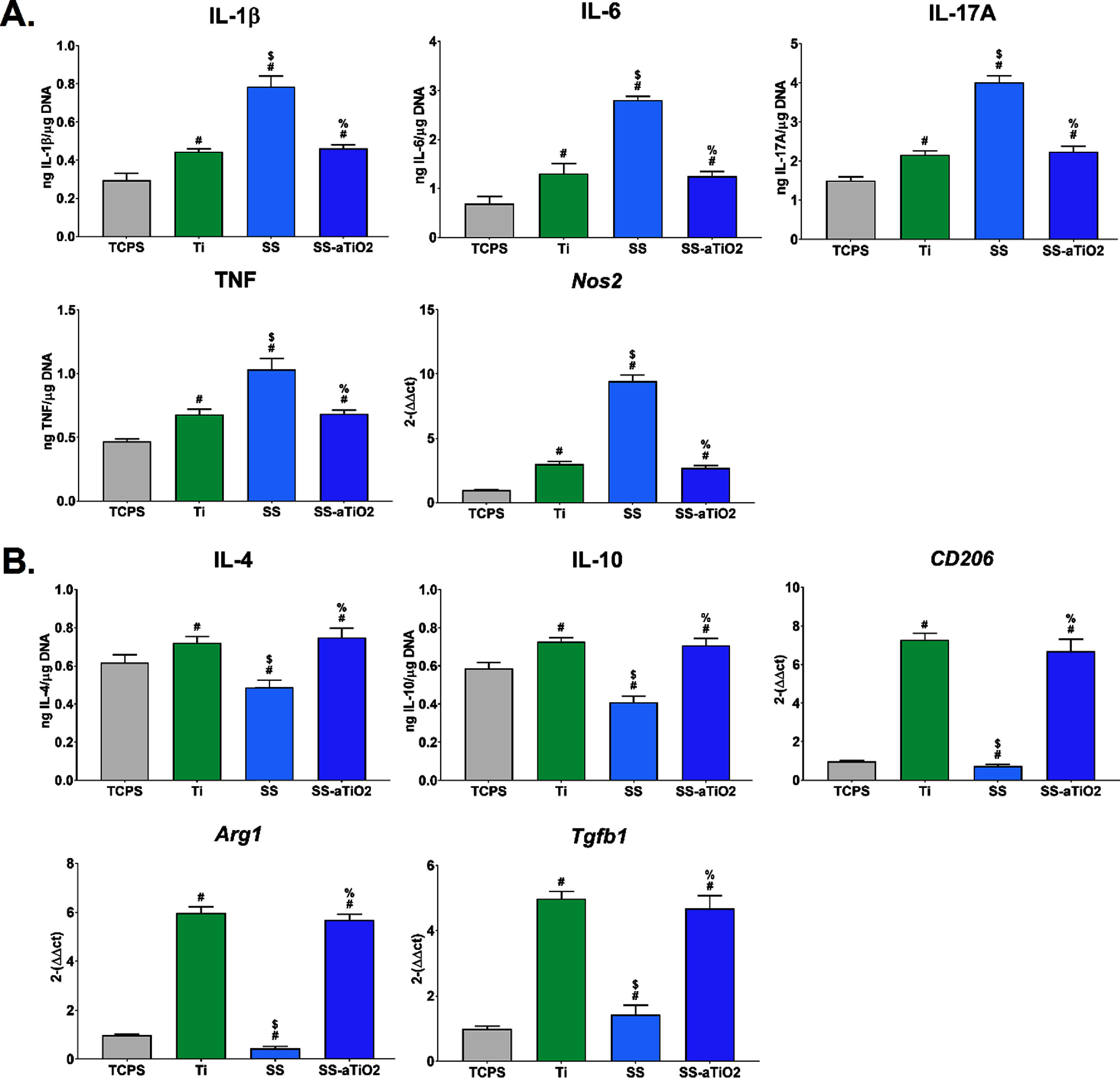
Macrophage inflammatory microenvironment after 24 h of culture on the surfaces. Tissue culture polystyrene (TCPS), sandblasted/acid-etched titanium (Ti), medical-grade stainless steel (SS), and SS substrate with amorphous Ti oxide nano-coating (SS-aTiO_2_). (A) Levels of pro-inflammatory markers. (B) Levels of anti-inflammatory markers. # *p* < 0.05, surfaces *vs* TCPS; $ *p* < 0.05, surfaces *vs* Ti; % *p* < 0.05, SS *vs* SS-aTiO_2_.

To characterize the effect of macrophage activity in response to changes in surface topography and chemistry, and consequently to assess the inflammatory ambient induced by the different surfaces studied, naïve macrophages were cultured on Ti, SS, SS-aTiO_2_, and TCPS as control. Macrophages produced significantly higher levels of pro-inflammatory cytokines (IL-1b, IL-6, IL-17A, TNF) and pro-inflammatory activation markers (Nos2) on SS in comparison to TCPS, Ti or SS-aTiO_2_. These levels were similar in cells cultured on SS-aTiO_2_ or Ti surfaces (figure 4(A)). In contrast, when studying the production of anti-inflammatory markers, macrophages production was similar between SS-aTiO_2_ or Ti and significantly larger than the number of anti-inflammatory activation markers (IL-4, IL-10, CD206, Arg1, Tgfb1) produced by macrophages cultured on SS or TCPS (figure 4(B)).

### 4.4. Bacterial colonization

**Figure 5.**
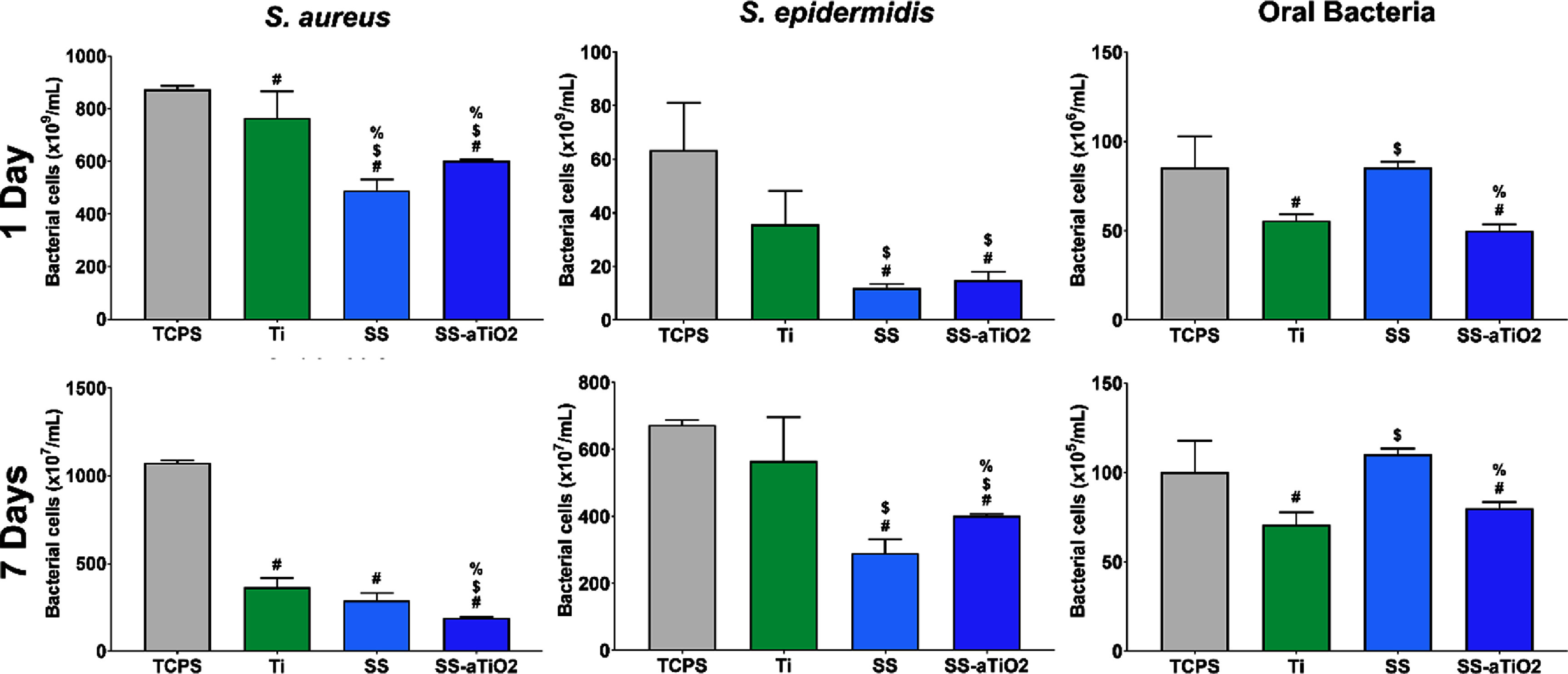
Bacterial colonization after 1 and 7 d of culture on the surfaces using *S. aureus*, and *S. epidermidis* (pure-culture model), and a consortium of eight oral species (co-culture model). # *p* < 0.05, surfaces *vs* TCPS; $ *p* < 0.05, surfaces *vs* Ti; % *p* < 0.05, SS *vs* SS-aTiO_2_.

Figure 5 presents the bacterial colonization on the experimental surfaces using the pure-culture model with two different strains and the co-culture model with the bacterial consortium after 1 and 7 d of culture for both culture models. In general, fewer bacterial cells colonized the test surfaces, SS, Ti, or SS-aTiO_2_, compared with the TCPS control. All test surfaces harbored a considerably lower number of bacterial cells after 7 d of incubation than after 1 d.

The number of *S. aureus* detected on the Ti, SS, and SS-aTiO_2_ surfaces was significantly lower than TCPS control in both incubation times; however, no significant differences in the number of bacteria between the experimental surfaces were observed. At 7 d of incubation, more *S. aureus* cells were detected on the uncoated SS and Ti surfaces compared to the nano-coated SS-aTiO_2_ surface (290, 365 and 190 × 10^7^ cells ml^−1^, respectively). On the other hand, *S. epidermidis* seemed to have less affinity for the test surfaces than *S. aureus* at least in the first hours of incubation, since a lower number of cells were detected after 1 d in all the test surfaces. However, at 7 d, the nano-coated SS-aTiO_2_ and uncoated SS surfaces significantly reduced the colonization of *S. epidermidis* (402 and 290 × 10^7^ cells ml^−1^, respectively) in comparison to the Ti surfaces (565 × 10^7^ cells ml^−1^).

Importantly, bacterial colonization was lower on the nano-coated SS-aTiO_2_ and Ti surfaces compared to the uncoated SS substrate and TCPS control in the oral bacterial consortium model at both incubation times. After 1 d of incubation, the nanocoated SS-aTiO_2_ surfaces showed the lowest bacterial colonization, followed by the Ti surfaces, being significantly lower than bacteria detected on the uncoated SS substrates (50, 55, and 85 × 10^6^ cells ml^−1^, respectively). Interestingly, the number of oral bacteria detected on the nanocoated SS-aTiO_2_ and Ti surfaces remained significantly lower than on the uncoated SS substrates after 7 d of incubation (80, 70 and 110 × 10^5^ cells ml^−1^, respectively). The SS surface showed the highest oral bacterial colonization over time.

## 5. Discussion

The magnetron sputtering method used in this study, allowed the coating of the SS substrates with a well adhered nano-layer of amorphous TiO_2_, without significantly modifying its topography, and therefore preserving its valuable bulk properties. Contrary to other techniques used for producing TiO_2_ coatings on SS, such as sol–gel spin coating [33], electrophoretic deposition [34], or dip-coating [35] methods, that can considerably modify the roughness and thickness of the substrate. Even more, most of the studies that report the coating of SS with TiO_2_ films, have been focused on improving its corrosion resistance and mechanical properties or for photocatalytic applications [36–39].

In the present work, an amorphous TiO_2_ nano-coating deposited on a non-Ti based metallic substrate, such as medical grade SS, demonstrated the potential to influence the cellular microenvironment for bone growth, affect the behavior of inflammatory cells, and reduce bacterial colonization of species related to oral and biomedical implant infections. The aTiO_2_ nano-coating was selected based on previous research, which showed that an amorphous TiO_2_ nanocoating deposited on Ti substrates enhanced the attachment and differentiation of various cell lines [29], resulting in greater expression of osteogenic proteins and reduced bacterial colonization compared to crystalline TiO_2_ nano-coatings and uncoated Ti surfaces [28, 40]. The biological functionality of the nanomodified SS surfaces was compared with uncoated SS substrates and sandblasted/acid-etched Ti surfaces, which were selected as comparative and positive controls, respectively. Titanium remains the most important material for manufacturing orthopedic devices [8, 41], and specifically, sandblasted/acid-etched Ti is widely used in commercially available dental implants [42].

When the aTiO_2_ nano-coating was deposited on the SS substrates, a significant increase in WCA values was observed. This increase is likely due to the hydrophobic nature of the aTiO_2_, as both SS and SS-aTiO_2_ surfaces exhibited similar surface roughness. The WCA values are determined by a complex interaction between the intrinsic surface energy of the material and its roughness [43–45]. According to Vogler’s definition {Vogler, 1998 #44), the CA limit separating hydrophilic from hydrophobic surfaces in a biological context is 65°. Thus, for a relevant biological context, the Ti and nano-coated SS-aTiO_2_ surfaces are considered hydrophobic, while the SS substrate surface exhibited a more hydrophilic character.

The hydrophobicity of biomaterial surfaces can be advantageous because it influences protein adsorption, a crucial factor in implant performance. Hydrophobic surfaces can adsorb more protein than hydrophilic ones [46], as the increase in system entropy due to water displacement from the surface favors protein adsorption through hydrophobic interactions [47]. Protein adsorption modulates surface properties and, consequently, subsequent cell attachment. This rapid and effective modulation by adsorbed proteins benefits biomaterials and has been effectively utilized in the market; most commercial implant surfaces are hydrophobic [48].

The nanocoated SS-aTiO_2_ surfaces preserved the surface topography and roughness of the bare substrates, while the comparative Ti surfaces were considerably rougher (approximately 0.75 μm). Different studies have reported that surface roughness influences the proliferation of MSC on Ti surfaces [49, 50]. Generally, higher roughness slightly inhibits cell proliferation, an effect observed when comparing the TCPS with the metallic rough surfaces. Nonetheless, our results showed that MSC proliferation, measured by DNA content, was similar between the three experimental surfaces.

Osteoblastic differentiation of MSCs varied between the experimental surfaces. MSCs cultured on the nanomodified SS-aTiO_2_ and Ti surfaces expressed very similar levels of the early osteoblastic differentiation markers (ALP and RUNX2), which were significantly higher than those expressed on the non-coated SS substrates. Even more, the levels of osteoprotegerin, a factor related to the inhibition of osteoclasts, and the levels of osteocalcin, a late marker of osteoblastic differentiation, were importantly increased on the SS-aTiO_2_ and Ti surfaces in comparison with the bare SS substrates. Therefore, the nanocoated SS-aTiO_2_ and the Ti surfaces committed the lineage of the MSCs cells towards an osteoblast lineage by activating the ‘master transcriptional regulator’ RUNX2. Once the RUNX2 is activated, the cells can be considered preosteoblasts, and they undergo osteoblastic differentiation, expressing molecular markers such as ALP and osteocalcin [51]. Similar results have also been previously reported for microstructured [52], nanostructured Ti surfaces [53], and other coatings like Ag_7_ZnO_3_HA using pre-osteoblastic cell lines [54]. Similarly, the levels of the VEGF (angiogenic factor) and the BMP-2 (osteogenic factor) were higher on the nanocoated SS-aTiO_2_ and Ti surfaces in relation to the levels observed on the non-coated SS substrates. This suggests the potential of the surfaces for favoring bone growth in the peri-material region, as it has been reported that VEGF expression enhances BMP-2-induced bone formation through modulation of angiogenesis [55]. On the contrary, the levels of the BMP-4 expression, another molecule of the transforming growth factor-beta superfamily, were reduced on the Ti and SS-aTiO_2_ surfaces in comparison with the levels on the SS substrate, possibly indicating a lessened capacity of the cellular microenvironment for stimulating osteogenesis [56]. In this regard, one study reports the unexpected decrease in BMP-4 associated with an increase in BMP-2 as a possible response to mechanical and/or cytokine stimulations in a murine model [57]. It is important to remark that in this work non-inductive molecules were used in the culture medium to promote stem cell differentiation or by external stimuli like irradiation of near-infrared, as other studies have previously done [58], so the bone microenvironment created by the MSC´s can be attributed mainly to the chemical composition of the aTiO_2_ nano-coating.

Macrophages are highly plastic innate immune cells that play a fundamental role in tissue healing after injury or trauma [59, 60]. It has been demonstrated that macrophages are sensitive to biomaterial surface properties such as topography and wettability [61, 62]. For Ti surfaces, it has been shown that hydrophilic surfaces preferentially activated macrophages into an anti-inflammatory phenotype while hydrophobic Ti surfaces skewed macrophages into a pro-inflammatory state [14, 63].

This study showed that macrophages cultured on SS surfaces increased their production of potent inflammatory cytokines such as IL-1*β*, TNF-*α*, and IL-17A, all associated with bone resorption and osteoclastic activation [64, 65]. Interestingly, macrophages cultured on SS modified with the aTiO_2_ nano-coating expressed reduced protein levels of the same inflammatory cytokines, with values like those of pure Ti surfaces. Furthermore, macrophage cultures on Ti or SS-aTiO_2_ increased gene expression of key markers of anti-inflammatory macrophages such as Arg1 and Mrc1 (CD206). Our results suggest that the initial adsorption of proteins (mediated by hydrophobic interactions) from the culture medium onto the biomaterial surface might be dictating the macrophage response.

In terms of bacterial colonization, surface properties played an important role. the roughness of the tested surfaces had a greater effect on the adhesion of *S. aureus*, a relevant aerobic opportunistic pathogen associated with implantable device infections [66]. This phenomenon could be explained in terms of the extra available surface of the samples; the rougher the surface, the larger the surface area, which confers more available space for the bacteria to adhere [67]. The Ti surfaces harbored more *S. aureus* than the SS-aTiO_2_ and SS surfaces, but no significant differences were detected between the nano-coated SS-aTiO_2_ surface and its bare SS substrate. This agrees with previous reports that described this strain as more sensitive to roughness than to the chemical composition of the surfaces [68]. Nevertheless, other studies have reported a major influence of the chemical composition on the colonization of *S. aureus* [69–71]. Regarding *S. epidermidis*, the number of bacterial cells detected on the Ti surface was significantly higher than on the SS and SS-aTiO_2_ surfaces. This suggests that *S. epidermidis* was more influenced by surface roughness and, to a lesser extent, by chemical composition. This is consistent with published results concluding that the adhesion of *S. epidermidis* on materials, including Ti and SS, is mostly influenced by surface roughness [72, 73]. In the present study, the colonization of *S. aureus* and *S. epidermidis* was mainly affected by surface roughness, showing more bacteria on the rougher Ti surfaces. In contrast, the adhesion of the consortium of anaerobic oral bacteria, which could be present in the microbiologic profile of peri-implantitis lesions [74], was more influenced by the chemical composition than surface roughness, showing similar levels of bacterial adhesion on Ti and SS-aTiO_2_ surfaces, which were significantly lower than those on SS surfaces. According to previously published data, some oral species, such as *E. corrodens* and *A. israelii*, are more sensitive to the chemical composition of the surface. Meanwhile, others are more sensitive to surface roughness, such as *P. intermedia* and *S. sanguinis* [75]. In this study, we found that the consortium of oral bacteria was significantly reduced on the SS-aTiO_2_ and Ti surfaces in relation to the bare SS substrate. This behavior could be ascribed to the chemical composition as well as the hydrophilic/hydrophobic character of the surfaces, since the non-Ti-based hydrophilic surface (SS substrate) presented higher bacterial colonization, as hydrophilic surfaces tend to harbor more bacteria than hydrophobic surfaces [48].

The bacterial test results showed that all experimental surfaces harbored fewer numbers of the oral bacteria consortium than the number detected of the single species. This phenomenon could be related to the difference in the average bacterial size and its relation to the bacteria-size/surface-area ratio. A previous work described an inverse relation between this ratio and the number of attached bacteria [28]. The average size of the oral bacteria consortium ranges from 2 to 8 μm, whereas the mean size of the *S. epidermidis* and *S. aureus* is 1 μm. Another possible explanation is the bacterial growth rate; aerobic bacteria commonly grow faster than anaerobic bacteria. The results obtained showed that the aTiO_2_ nano-coating on SS substrates with tailored roughness seems to have the potential to decrease the bacterial adhesion and, consequently, the pathogenic biofilm formation on oral implant devices, besides implantable orthopedic materials with nanocoating and tailored surface.

## 6. Conclusions

The deposition of an aTiO_2_ nano-coating on medical-grade SS substrates via magnetron sputtering has been shown to maintain the original surface topography and roughness while remarkably enhancing the biological properties. Our analyses indicate that while surface roughness and wettability modestly influence MSC proliferation, the chemical composition of the Ti and SS-aTiO_2_ surfaces significantly promotes MSC differentiation into osteoblastic lineages and boosts the production of proteins crucial for bone regeneration, an effect notably absent in uncoated SS substrates.

Furthermore, macrophages cultured on the aTiO_2_ nanocoated surfaces foster a comparable anti-inflammatory microenvironment to those cultured on pure Ti surfaces, in contrast to the pro-inflammatory response observed on the uncoated SS surfaces. This suggests the potential of aTiO_2_ nano-coatings to beneficially modulate the *in vivo* inflammatory responses.

Importantly, the aTiO_2_ nano-coating significantly influences bacterial colonization dynamics. The smaller roughness of SS, whether coated or uncoated, generally reduced colonization by *S. aureus* and *S epidermidis*, particularly when compared to the rougher Ti surfaces. Additionally, the unique chemical composition and wettability of the Ti and SS-aTiO_2_ surfaces appear to deter colonization by oral anaerobic bacterial consortia, indicating an added antimicrobial benefit.

The strategic application of aTiO_2_ nano-coatings on non-Ti metallic surfaces, particularly with controlled roughness, emerges as a promising approach not only to enhance osseointegration and promote an anti-inflammatory microenvironment but also to decrease pathogenic bacterial adhesion and biofilm formation on metallic biomedical devices. This functional capability positions of aTiO_2_ nano-coatings represents an important advancement in the design and processing of SS materials for next-generation of orthopedic and dental implants.

## Data Availability

All data that support the findings of this study are included within the article (and any supplementary files).
